# Array of nanosheets render ultrafast and high-capacity Na-ion storage by tunable pseudocapacitance

**DOI:** 10.1038/ncomms12122

**Published:** 2016-06-30

**Authors:** Dongliang Chao, Changrong Zhu, Peihua Yang, Xinhui Xia, Jilei Liu, Jin Wang, Xiaofeng Fan, Serguei V. Savilov, Jianyi Lin, Hong Jin Fan, Ze Xiang Shen

**Affiliations:** 1 School of Physical and Mathematical Sciences, Nanyang Technological University, Singapore 637371, Singapore; 2 State Key Laboratory of Silicon Materials, Department of Materials Science and Engineering, Zhejiang University, Hangzhou 310027, China; 3 Energy Research Institute @ NTU, Nanyang Technological University, Singapore 639798, Singapore; 4 College of Materials Science and Engineering, Jilin University, Changchun 130012, China; 5 Department of Chemistry, Moscow State University, Moscow 119992, Russia

## Abstract

Sodium-ion batteries are a potentially low-cost and safe alternative to the prevailing lithium-ion battery technology. However, it is a great challenge to achieve fast charging and high power density for most sodium-ion electrodes because of the sluggish sodiation kinetics. Here we demonstrate a high-capacity and high-rate sodium-ion anode based on ultrathin layered tin(II) sulfide nanostructures, in which a maximized extrinsic pseudocapacitance contribution is identified and verified by kinetics analysis. The graphene foam supported tin(II) sulfide nanoarray anode delivers a high reversible capacity of ∼1,100 mAh g^−1^ at 30 mA g^−1^ and ∼420 mAh g^−1^ at 30 A g^−1^, which even outperforms its lithium-ion storage performance. The surface-dominated redox reaction rendered by our tailored ultrathin tin(II) sulfide nanostructures may also work in other layered materials for high-performance sodium-ion storage.

## Abstract (web summary)

Sodium ion batteries are a promising alternative to lithium ion technology, however their sluggish sodiation kinetics currently hinder performance. Here the authors fabricate ultrathin layered tin(II) sulfide nanostructures displaying a pseudocapacitance contribution for high capacity sodium ion anodes.

## Graphical abstract

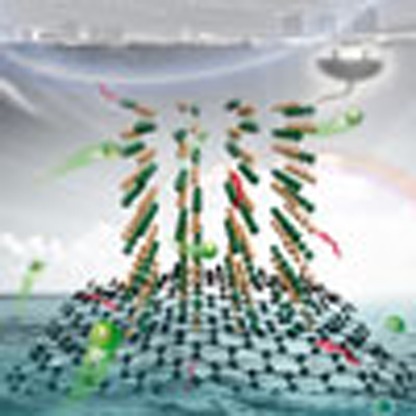

While open framework materials have proven effective in constructing kinetically favourable sodium (Na)-ion channels as cathodes for sodium-ion batteries (SIBs)^1^,^2^,^3^,^4^,^5^,^6^,^7^, the sluggish Na-ion transport and severe volume expansion currently limit the rate performance and stability in most anode materials. The typical alloying materials (for example, Sn, Ge, Pb, Sb) possess high capacities (Na_15_Sn_4_: 847 mAh g^−1^, Na_3_Ge: 1,108 mAh g^−1^, Na_15_Pb_4_: 484 mAh g^−1^, Na_3_Sb: 660 mAh g^−1^) for Na storage but have severe volume expansion/contraction during the Na alloying/dealloying (∼360−420%) (refs 8, 9, 10). To address this pulverization issue, one effective approach is to design integrated electrodes in which nanosized active materials are grafted to a secondary matrix^8^,^10^. Compared with tin (Sn) metal anodes, tin-based oxides and chalcogenides can store Na^+^ through a combined electrochemical conversion and alloying mechanisms, giving rise to higher theoretical capacities (SnO_2_: 1,378 mAh g^−1^, SnS_2_: 1,136 mAh g^−1^, SnS: 1,022 mAh g^−1^) (ref. 11). Sulfides are typically more reversible than oxides due to relatively weaker M−S ionic bonds compared with M–O bonds, resulting in kinetically more favourable and higher first-cycle efficiency of tin chalcogenides^11^. Moreover, with merits of high electrical conductivity (0.193−0.0083 S cm^−1^), higher capacity and earth abundancy, tin(II) sulfide (SnS) is considered to be a very promising anode material for SIBs. SnS has a smaller lattice expansion (242%) in the sodiation/desodiation process than SnS_2_ (324%) (ref. 11). This correlates to a two-structure phase reaction in SnS (from orthorhombic-SnS to cubic-Sn to orthorhombic-Na_15_Sn_4_) compared with the three-structure transformation in SnS_2_ (from hexagonal-SnS_2_ to tetragonal-Sn to orthorhombic-Na_15_Sn_4_ (ref. 11). Despite the high capacities in Sn-based alloying materials, high rate capability and fast-charging have not yet been reported, to the best of our knowledge.

Compared with the diffusion-controlled process (insertion, conversion and alloying) in conventional Li/Na-ion storage battery materials, capacitive charge storage has the advantage of rendering high charging rate and therefore high power. In particular, pseudocapacitance refers to underpotential deposition, faradaic charge-transfer reactions including surface or near-surface redox reactions and bulk fast ion intercalation^12^,^13^,^14^,^15^,^16^. Pseudocapacitance can be intrinsic or extrinsic to a material^12^; intrinsic ones (such as RuO_2_, MnO_2_ and Nb_2_O_5_) display the capacitive characteristics for a wide range of particle sizes and morphologies, whereas extrinsic ones (such as LiCoO_2_, MoO_2_ and V_2_O_5_) emerge only when they are made into nanoscale dimensions to maximize reaction sites on the surface^14^,^15^,^16^,^17^. So far, enhanced pseudocapacitive contributions have been realized in some insertion and conversion Li-ion battery (LIB) and SIB materials with high-rate performance (see summary in Supplementary Table 1)^12^,^18^,^19^,^20^. However, it has not yet been implemented in alloying-type materials where the challenge is to realize high-capacity materials that can accommodate fast kinetics.

Here we demonstrate and prove by quantitative kinetics analysis, the pseudocapacitive contribution to the high capacity of Na-ion storage in few-layered SnS nanosheet arrays directly grown on a graphene foam (GF) backbone (Fig. 1). To the best of our knowledge, our purposely engineered SnS nanohoneycomb structure exhibits the highest reversible capacity, rate capability compared with the reported carbon allotrope, metal/alloy and metal oxides/sulfides as SIB anodes. Excitingly, due to the maximized pseudocapacitive contribution, its rate performance in Na-ion storage even exceeds that for Li ion. Our result may bring a paradigm shift in SIB anode materials to layered metal sulfides, and also afford deeper understanding as well as other nanoscale engineering strategies to boost the performance of SIBs.

## Results

### Structure and growth mechanism

**Figure 1:**
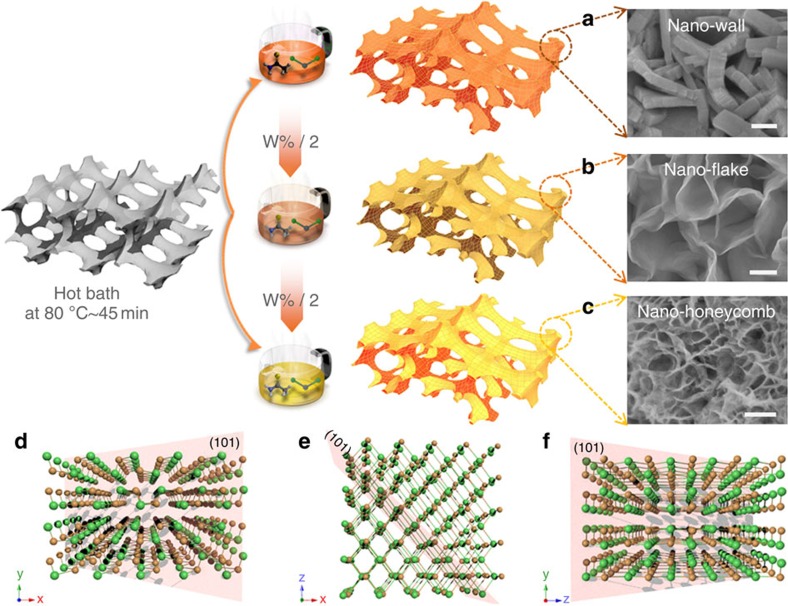
Synthesis and structure of SnS nanostructures. (**a**–**c**) SnS nanostructures synthesized in different solution concentrations for (**a**) nano-wall; (**b**) nano-flake; (**c**) nano-honeycomb. Scale bar, 200 nm. (**d**–**f**) Schematic illustrations of the SnS laminar structure viewed along the [001], [010] and [100] zone axis with inserted (101) planes.

Figure 1a–c illustrates the fabrication procedure of the flexible GF-supported SnS electrodes by a rapid one-step *in situ* hot bath route (details are described in the Methods section). To demonstrate the morphology-dependent property, we obtained three types of samples with gradient morphologies that are denoted as nanowall (NW), nanoflakes (NF) and nanohoneycomb (NH). Herein, it is important to control the precursor concentration and nucleation rates to achieve desirable SnS nanostructure. Our synthesis leads to a full and uniform coverage of the substrate by SnS nanostructures, for which the size distribution can also be tailored (Supplementary Fig. 1). The SnS presents average lateral sizes of 400–500, 400–500 and 50–70 nm and thickness of ∼150, 10 and 5 nm for NW, NF and NH, respectively. The growth of the SnS nanostructures is also proved substrate friendly (Ni foam, carbon cloth and ITO, see Supplementary Fig. 2). A growth mechanism is proposed based on the crucial roles of the substrate and ethanol, which involves (i) the hydrolysis of thioacetamide and (ii) the *in situ* metathesis reactions, self-assembly and oriented crystallization processes (details are provided in Supplementary Fig. 3 and Supplementary Note 1)^5^. The structure evolution and purity are verified by X-ray diffraction , energy-dispersive spectroscopy and Raman measurements (Supplementary Figs 4 and 5). The surface chemical-bonding state of GF-SnS electrode is also detected by X-ray photoelectron spectroscopy (XPS) and presented in Supplementary Fig. 6. The existence of C–S bonds is confirmed by spectra of both S 2p (163.7 eV) and C 1s (285.7 eV). XPS results suggest that the SnS might be chemically bonded with the GF matrix besides physical deposition^19^ (details are provided in Supplementary Note 2).

**Figure 2:**
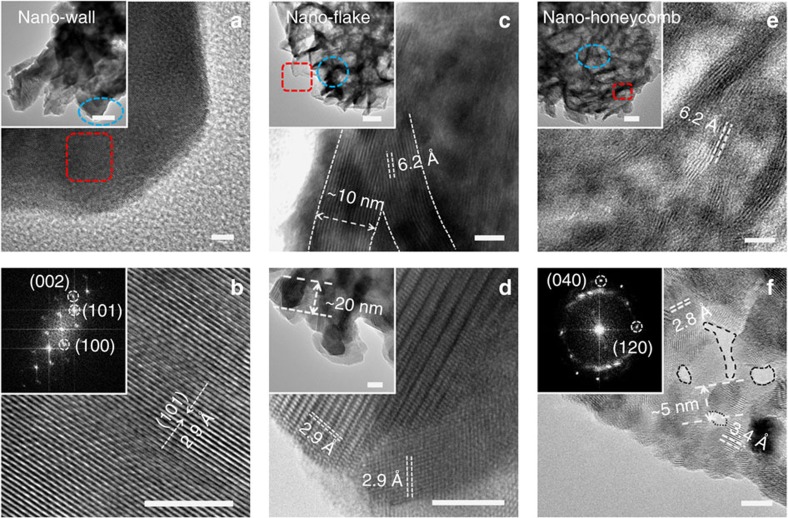
TEM images of SnS nanostructures. (**a**,**b**) HRTEM images for the nanowall SnS structure. Scale bar, 5 nm. Inset a: low magnification TEM image (scale bar, 100 nm) and (**b**) fast Fourier transform (FFT) pattern along [010] zone axis. (**c**,**d**) HRTEM images for nanoflake SnS structure. Scale bar, 5 nm. Insets **c**,**d**: low magnification TEM images. Scale bars of 400 and 10 nm, respectively. (**e**,**f**) HRTEM images for nanohoneycomb SnS structure. Scale bar, 5 nm. Inset **e**: low magnification TEM image. Scale bar, 30 nm. Inset **f**: FFT pattern in the [001] zone axis. The dashed loops denote the mesopores.

Transmission electron microscopy (TEM) and high-resolution transmission electron microscopy (HRTEM) images in Fig. 2 further confirm the crystallographic orientation and unique nanosheet-on-microstructure three-dimensional (3D) porous nanowall, nanoflake and nanohoneycomb architectures. For SnS NW (Fig. 2a,b), the lattice-resolved HRTEM image shows interplanar spacing of 2.9 Å for the (101) planes of SnS. Further, the inset fast Fourier transform spots also reveal the existence of (101), (002) and (100) facets in the [010] zone axis, demonstrating that the layers of SnS are stacked along the [010] direction (see illustration in Fig. 1e). For SnS NF (Fig. 2c,d), the interconnected nanoflakes are regular with a periodic stacking of fringes (∼15 layers) enforced by van der Waals interactions along the [010] direction. The layer distance is measured as ∼6.2 Å, which is slightly larger than the layer-to-layer spacing in reported work^21^. The lateral view of the nanoflake in Fig. 2d also illustrates an interplanar spacing of 2.9 Å for the (101) planes of SnS with a crystal grain size ∼20 nm. The results also confirm the formation of nanosheets by stacking of (010) facets. Similarly, SnS NH also presents intrinsic corrugations and lamellar structure in the nanosheets with interlayer spacing ∼6.2 Å, but with thinner thickness (∼6 layers). Interestingly, numerous tiny nanoclusters (∼5 nm) and nanocavities (3–5 nm) are also observed from the cross-section of the wrinkles in Fig. 2f. Meanwhile, HRTEM and fast Fourier transform pattern reveals clear lattices with spacings of 3.4 and 2.8 Å for (120) and (040) planes, respectively, under [001] zone axis. A schematic illustration of the SnS laminar structure seen from [001] zone axis can be seen from Fig. 1d.

### Na-ion storage performance

Our designed battery electrodes allow electron and sodium-ion transfer through the GF–SnS network without the necessity of extra binders, conductive additives or metal Cu current collectors, which are essential for exploring intrinsic sodium-storage properties of active material and increasing the energy/power densities of the full cell^5^,^22^. Herein, GF serves as both a lightweight 3D porous current collector (for electron transfer) and compressible/flexible backbone. The porous nanoarray feature prevents the aggregation and expansion of SnS during charge/discharge cycles. During sodiation, electrolyte can enter the interval between nanoarrays on both outside and inner surface of GF, so that the Na ion and electrons can react with the SnS nanoarrays effectively.

The reaction of SnS with sodium undergoes two steps: conversion followed by alloying according to equations (1) and (2) (ref. 11):


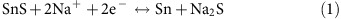



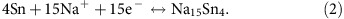


**Figure 3:**
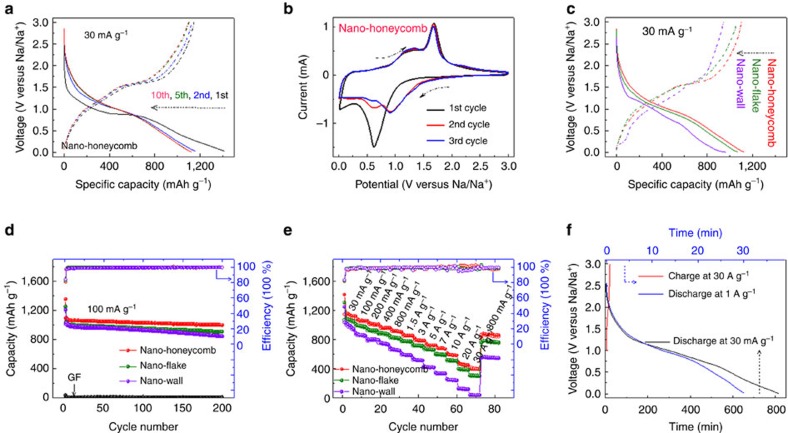
Electrochemical Na^+^ storage performance of SnS electrodes. (**a**) Galvanostatic charge/discharge profiles during the first 10 cycles of the nanohoneycomb electrode. (**b**) CV curves of the first three cycles of the SnS nanohoneycomb electrode at a scan rate of 0.2 mV s^−1^. (**c**) Galvanostatic charge/discharge profiles of SnS electrodes after five cycles activation. (**d**) Long-term cycling performances of SnS electrodes and the pure graphene foam electrode at a current density of 100 mA g^−1^. (**e**) Rate performances of SnS electrodes at various current densities from 30 to 30,000 mA g^−1^. (**f**) Fast charging (charge at 30 A g^−1^ in 1 min, discharge at 30 mA g^−1^ and 1 A g^−1^ with ∼13 h and 30 min, respectively) properties of the nanohoneycomb electrode.

From the galvanostatic discharge–charge results (Fig. 3a and Supplementary Fig. 7), the first discharge presents a steep decrease and remains relatively flat in voltage. This may be due to the following two possible reasons: (1) deactivated surface and related high electrochemical polarization during the first sodiation; (2) transition from crystalline SnS to metal Sn nanograins and Na_2_S (equation (1)) (refs 11, 23). However, the subsequent curves after the first three cycles almost overlap, indicating a stable surface state, structure and electrochemical reversibility after the initial activation process. The detailed reaction processes could be disclosed by the cyclic voltammetric (CV) curves (Fig. 3b). In the first cathodic scan, two prominent peaks were observed at around 0.6−0.7 and 0.01−0.1 V, respectively. The former is associated with both the conversion reaction (equation (1)) and the alloying reaction (equation (2), Na_*x*_Sn, *x* ∼0.75) because it is difficult to distinguish the conversion and alloying peaks especially in the first discharge process^11^,^24^. Obviously this peak also includes the contribution from solid electrolyte interphase (SEI) formation since its intensity significantly decays in the subsequent cycles. The peak at 0.01−0.1 V is regarded as the reaction between Na and Na_*x*_Sn (*x* ∼3.75) alloy due to the multi-step Na–Sn alloying feature^11^. In the anodic scan, a serial of small peaks below 1.4 V (∼0.3, 0.7, 1.2−1.4 V) correspond to the multi-step dealloying reaction of Na_*x*_Sn (ref. 24); whereas the distinct peak at ∼1.7 V could be attributed to the reversible conversion reaction from Sn to SnS, which was also observed in SnS@G^11^, 3D porous interconnected SnS (ref. 25), and proved by *ex situ* Raman results of the electrodes after charging and discharging processes (Supplementary Fig. 8).

The first sodiation and desodiation capacities of GF–SnS NH are 1,416 and 1,147 mAh g^−1^, respectively, calculated based on active materials. A rather high first-cycle coulombic efficiency of >80% is observed. A reversible capacity more than 1,100 mAh g^−1^ could be maintained in the following cycles. We note this value is higher than all reported ones from sodium alloys, oxides, sulfides and carbonaceous anodes so far (see comprehensive comparison in Supplementary Table 2), to the best of our knowledge. Note that the pure GF contributed negligibly to the capacity: ∼30 mAh g^−1^ for the first discharge process and <10 mAh g^−1^ in the following cycles. Compared with NH electrode, NF presents a similar behaviour due to their similar microtopography, whereas the NW one exhibits a suppressed capacity and higher polarization. More importantly, the voltage profile in NW electrode shows more obvious plateaus, which is a typical feature of diffusion-controlled charge storage of battery materials^16^. The improvements in the coulombic efficiency and high reversible capacity of GF–SnS NH electrode should be correlated to the reversible formation/decomposition of the polymeric film on the surface of SnS NH (refs 11, 26), its distinct disorder two-dimensional structure, ultrathin-layered mesoporous SnS nanocrystals, and as a result, its unique electrochemical mechanism (capacitive contribution), which is to be discussed below. The GF–SnS electrodes deliver excellent capacity retention from the third cycle onwards. After 200 cycles, the capacity retains at 1,010 mAh g^−1^ for the NH electrode with well-preserved microstructure (Supplementary Fig. 9).

The rate capability is a crucial indicator for large scale application of batteries, such as regenerative braking and fast recharging of electric vehicles and cellphones. The drawback of low power becomes particularly evident in high capacity (that is, high energy density) materials^14^. It is found that the NH electrode has the best rate capability, in addition to consistently highest capacity, among the three GF–SnS electrodes (see Fig. 3e). For a 1,000-fold increase in current density (from 30 to 30 A g^−1^), a discharge capacity of more than 400 mAh g^−1^ (in 1 min) could still be retained. If this electrode were used to power a cellphone system, it is estimated that the battery could be charged in 1 min and discharged in ∼13 h at 30 mA g^−1^ (Fig. 3f). On the basis of a comprehensive summary (Supplementary Table 2), this is the best rate capability among all reported anode materials for SIBs, to the best of our knowledge. The preliminary result of full-cell fabrication (see Supplementary Fig. 10, Na_3_(VO)_2_(PO_4_)_2_F cathode\\SnS anode) demonstrates the potential commercial application of our SnS electrodes to be considered as an anode material for SIBs, although further optimization is urgent to improve the skill in full-cell fabrication and cycling stability.

### Kinetics and quantitative analysis

**Figure 4:**
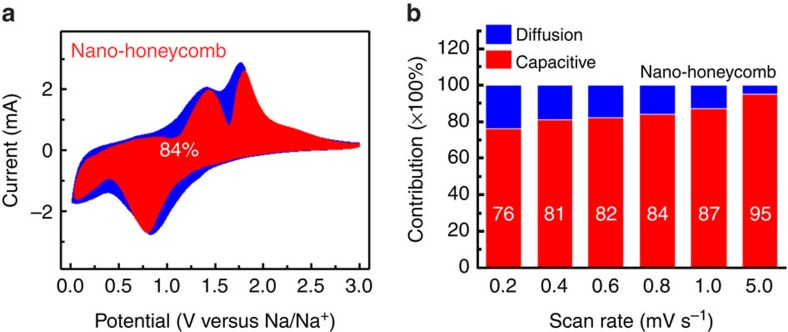
Kinetics and quantitative analysis of the Na^+^ storage mechanism. (**a**) Capacitive (red) and diffusion-controlled (blue) contribution to charge storage of nanohoneycomb at 0.8 mV s^−1^. (**b**) Normalized contribution ratio of capacitive (red) and diffusion-controlled (blue) capacities at different scan rate.

To explain the high-rate performance, we analysed the redox pseudocapacitance-like contribution in the GF–SnS electrodes by investigating the kinetics of the SnS electrodes (Fig. 4) to separate the diffusion-controlled capacity and capacitive capacity^15^,^27^. Resulting from the stepwise sodiation mechanism, CV curves with similar shapes at various scan rates from 0.2 to 0.8 mV s^−1^ (Supplementary Fig. 11) display two broad cathodic peaks as the scan rate increases. As cation intercalation reaction can be ruled out from our SnS electrode, we mainly consider the below three charge-storage mechanisms: the diffusion-controlled faradaic contribution from conversion and alloying reaction, the faradaic contribution from charge transfer with surface/subsurface atoms (that is, extrinsic pseudocapacitance effect), and the non-faradaic contribution from electrical double-layer effect^17^,^19^,^20^.

The ratios of Na-ion capacitive contribution can be further quantitatively quantified by separating the current response *i* at a fixed potential *V* into capacitive effects (proportional to the scan rate *v*) and diffusion-controlled reactions (*k*_2_*v*^1/2^), according to Dunn^15^,^28^:


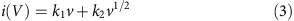


By determining both *k*_1_ and *k*_2_ constants, we can distinguish the fraction of the current from surface capacitance and Na^+^ semi-infinite linear diffusion. Fig. 4a shows the typical voltage profile for the capacitive current (red region) in comparison with the total current. A dominating capacitive contribution (∼84%) is obtained for the NH electrode. As the scan rate increases, the role of capacitive contribution further enlarges (Fig. 4b) with a maximum value of ∼95% at 5 mV s^−1^. By similar analysis, the pseudocapacitive contribution is found more than 80% for the NF electrode, but only around 60% for the NW one at 0.8 mV s^−1^ (Supplementary Figs 12 and 13). This is unsurprising since the pseudocapacitive contribution should play a critical role for smaller particle size with high surface area (∼154 m^2^ g^−1^) and/or high porosity (mesoporous from 7 to 37 nm, see Supplementary Fig. 14)^15^,^18^. Finally, the thin-film electric conductivity (Supplementary Fig. 15) and electrochemical impedance spectra (Supplementary Fig. 16) suggest that the NH electrode have a favourable charge transfer kinetics compared with NW electrode.

### Comparison with Li-ion storage capability

It is widely believed that Na^+^ transport and storage are more sluggish with more severe lattice expansion than the Li^+^ one because of the larger radius of Na ions^1^,^29^,^30^,^31^,^32^. So far, the performance in SIB is generally worse than that in LIBs when the same electrode material is used, including capacity, high rate capability and polarization. Herein, we carefully compare the performance of SnS NH anode for both Na^+^ and Li^+^ tests.

**Figure 5:**
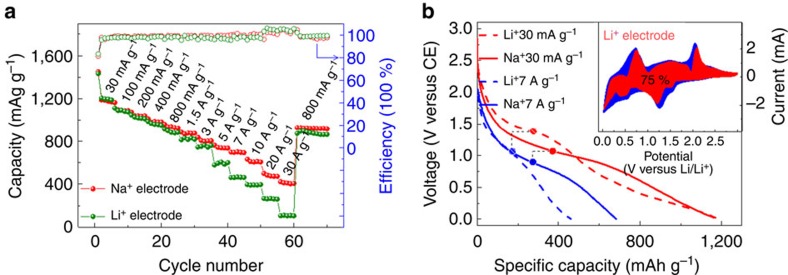
Comparison between Na-ion and Li-ion storage. (**a**) Rate performance comparison of the nanohoneycomb electrode at various current densities from 30 to 30,000 mA g^−1^. (**b**) Galvanostatic profiles of Na, Li electrodes at rates of 30 mA g^−1^ and 7 A g^−1^ after activation. Inset: capacitive (red) and diffusion-controlled (blue) contribution to charge storage at 0.8 mV s^−1^ during Li uptake. All of the batteries were tested in the same voltage range of 0.01–3 V versus Na/Na^+^ and Li/Li^+^ for Na-ion and Li-ion batteries, respectively.

To avoid the influence on Li uptakes in GF backbone, in our comparison experiment we used the SnS NH grown on Ni foam as the same electrode. Figure 5 shows the results. Strikingly, one can see the rate capacity for sodiation/desodiation is superior to Li^+^ uptakes, particularly in the high-rate regions. At current of 30 A g^−1^, the electrode delivers a Na^+^ discharge capacity of ∼ 410 mAh g^−1^ compared with ∼105 mAh g^−1^ for the Li^+^ electrode. In the galvanostatic charge/discharge processes (Fig. 5b), the Li^+^ electrode shows two distinct plateaus at 1.2−1.4 and 0.01−0.4 V versus Li/Li^+^ corresponding to their CV curves (Supplementary Fig. 17a), suggesting a lower fraction of capacitive contribution. The first discharge plateau during lithiation is attributed to the conversion reaction from SnS→Sn and the second one is the alloying reaction-forming Li_15_Sn_4_ phase^33^,^34^. The polarization from 30 mA g^−1^ to 7 A g^−1^ during lithiation (∼340 mV) is twice to that in sodiation (∼170 mV). In addition, the capacitive fraction for Li^+^ storage is 75% (inset in Fig. 5b), whereas 85% for the Na^+^ storage of the same electrode (Supplementary Fig. 18). Similar higher Na^+^ capacitive contribution than that of Li^+^ had also been observed in Li_4_Ti_5_O_12_ spinel thin film electrode but with much lower discharge capacity^20^. Finally, the sodiation discharge curves have more moderate and continuous operation voltage than the lithiation ones (Fig. 5b), which is favourable to achieving high energy density of full cells and avoiding dendrite growth^19^.

## Discussion

**Figure 6:**
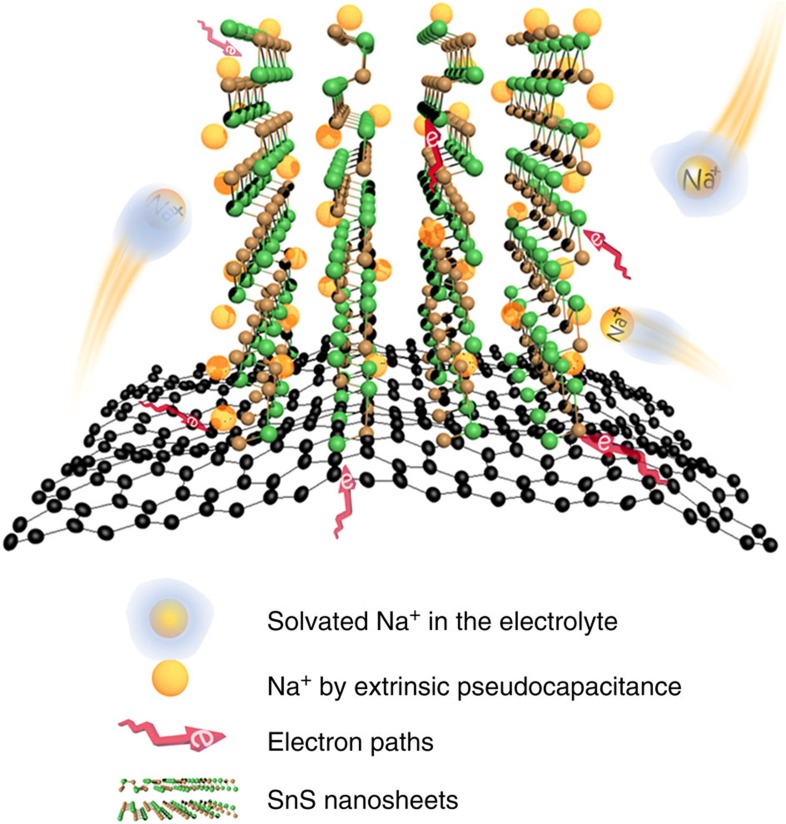
Schematic illustration of high-rate charge storage of SnS architecture. During transfer of ions and electrons, the solvated Na^+^/electron can easily enter into the open spaces between neighbouring ultrathin nanosheets on both the outside and inner surface of the graphene foam. After de-solvation of Na^+^ on the surface of the layered SnS architecture, rapid sodiation takes place by the surface-dominated extrinsic pseudocapacitance.

The results presented above demonstrate sodium-ion storage with both high capacity and high rate capability rendered by tunable extrinsic pseudocapacitance in our GF-supported SnS nanosheets. The electrode architecture provides, to the best of our knowledge, the highest reported reversible capacity of 1,100 mAh g^−1^ at 30 mA g^−1^. Even at a high current density of 30 A g^−1^ (1,000-fold increase), the capacity is retained at 400 mAh g^−1^, which is higher than that of Li^+^ electrode (∼105 mAh g^−1^ at 30 A g^−1^). As the diffusion time of ions (*t*) is proportional to the square of the diffusion length (*L*), *t* ≈ *L*^2^/*D*, a short lithium diffusion time of ∼0.01 s is obtained on the basis of the ultrathin SnS architectures. As a consequence, similar to supercapacitors, the limiting factor for high rate charge/discharge is the transfer of ions and electrons to the surface of nanosheets rather than the conventional solid-state diffusion. As schematically shown in Fig. 6, the strongly solvating groups (carbonyl groups) in the organic electrolyte will arrange in an appropriate manner as a preferred solvation shell of the cations (Li^+^ or Na^+^) (refs 35, 36, 37), and it has been proven that sodium ion presents a weaker solvation shell with smaller de-solvation energy and lower activation barrier for sodiation transport compared with the lithium ion^35^,^38^. In additionally, higher mobility and conductivity of Na^+^ solutions also contribute to the ion transfer in the electrolyte^39^,^40^. As a result, faster sodiation/desodiation kinetics are possible for SIBs as compared with LIBs^35^,^39^,^40^,^41^.

The surface-dominated extrinsic pseudocapacitance is identified as a major energy-storage mechanism in favour of high capacity and fast Na^+^ uptakes (Fig. 6). First, the chemically bonded GF–SnS hybrid demonstrates excellent structure stability and electronic/ionic conductivity through the network, which have also been shown to be a prerequisite for the extrinsic pseudocapacitance in nanosized MoO_2_ (ref. 16). Moreover, the nanoscale dimension, especially thickness of the electrode materials, has been emphasized to be an important factor on the rate properties and corresponding redox capacitive contribution^20^,^27^,^42^,^43^. Compared with the SnS NW, the few layered architecture and mesoporous iso-oriented nanocrystals nature of the NH enables an interior Na^+^ or electrolyte access into the van der Waals gaps of nanosheets, and thus results in both the exterior and interior parts participating in the electrochemical reaction^15^. This feature also facilitates ion access and shortens the ion diffusion.

Our kinetics analysis verifies the surface-dominated redox reaction mechanism in the Na-ion storage process, whereas the battery-type diffusion contribution is suppressed. This is closely correlated to the engineered thin-sheet structure of SnS NH, and may account for the superior Na^+^ storage performance (high rate capacity) of the SnS NH to its Li^+^ one. This strategy renders increased power density with maintained high energy density. This encouraging result may accelerate further development of SIBs by smart nanoengineering of the electrode materials.

## Methods

### Synthesis and characterization

SnS nanostructures were fabricated by a facile hot bath method. First, precursors with three sets of different concentrations, namely, high, medium and low concentrations of tin(II) chloride dehydrate: thioacetamide, respectively for nanowall (100:300 mM), nanoflake (50:150 mM) and nanohoneycomb (25:75 mM), were dissolved in 50 ml ethanol at 80 °C. Then 3D GFs(2 × 5 cm^2^, ∼0.8 mg cm^−2^, prepared by chemical vapour deposition method according to our previous result^44^) or other type of substrates such as Ni foam, carbon cloth and ITO glass were immersed into the above reaction solutions and kept for 45 min. Finally, the samples were collected and rinsed with distilled water and ethanol in turn three times, and dried at 150 °C in vacuum to obtain 3D GF-supported SnS free-standing electrodes. The SnS loading was ∼1.0 mg cm^−2^ for nanoflake and nanohoneycomb electrode, and 1.2 mg cm^−2^ for nanowall electrode.

The crystal structures of the samples were identified using X-ray diffraction (RigakuD/Max-2550 with Cu-Kα radiation). Raman spectra were obtained with a WITec-CRM200 Raman system (WITec, Germany) with a laser wavelength of 532 nm (2.33 eV). The morphologies of the samples were characterized by field emission scanning electron microscopy. The structures of the samples were investigated by HRTEM(JEOL JEM-2010F at 200 kV). The XPS measurements were performed by a VG ESCALAB 220i-XL system using a monochromatic Al Ka1 source (1,486.6 eV). The thin-film electric conductivity was measured by four-point probe sheet resistance. The surface area of the SnS electrode was determined by N_2_ adsorption/desorption isotherms.

### Electrochemical measurements

Standard CR2032-type coin cells were assembled in an argon-filled glove box (Mbraun, Germany) with the as-fabricated GF-supported SnS nanoarrays as the working electrode (without any binder or additives). For SIB fabrication, the metallic sodium foil as the counter-electrode, 1 M NaPF_6_ in ethylene carbonate (EC)–diethyl carbonate (DEC)–fluoroethylene carbonate (FEC) (1:1:0.03 in volume) as the electrolyte, and glass fibre as the separator. For the LIB case, except for the metallic lithium foil as the counter-electrode and 1 M LiPF_6_ as the solute, the other parameters are the same with the SIB fabrication. For full-cell testing, the cathode-active material was Na_3_(VO)_2_(PO_4_)_2_F nanoparticle. The one-time discharge/charge cycled SnS NH served as the anode. The weight ratio between anode and cathode active material was ∼0.15:1. The specific capacity was calculated based on the mass of the cathode-active material. The CV measurements were carried out using CHI660 electrochemical workstation. Electrochemical impedance spectroscopy was recorded on Solartron 1470E, the amplitude of the sine perturbation signal was 5 mV, and the frequency was scanned from the highest (10 kHz) to the lowest (5 mHz). Galvanostatic charge discharge cycles were tested by Neware battery tester at different current densities at room temperature.

### Data availability

The authors declare that the data supporting the findings of this study are available within the article and its Supplementary Information files.

## Additional information

**How to cite this article:** Chao, D. *et al.* Array of nanosheets render ultrafast and high-capacity Na-ion storage by tunable pseudocapacitance. *Nat. Commun.* 7:12122 doi: 10.1038/ncomms12122 (2016).

## Supplementary Material

Supplementary Information Supplementary Figures 1-18, Supplementary Tables 1-2, Supplementary Notes 1-2 and Supplementary References.
