# Supplementary material for: Array of nanosheets render ultrafast and high-capacity Na-ion storage by tunable pseudocapacitance
Source: Nat Commun. 2016 Jun 30;7:12122. doi: 10.1038/ncomms12122 (PMC4931321; doi:10.1038/ncomms12122)
Supplement: Supplementary Information — Supplementary Figures 1-18, Supplementary Tables 1-2, Supplementary Notes 1-2 and Supplementary References. [file ncomms12122-s1.pdf]

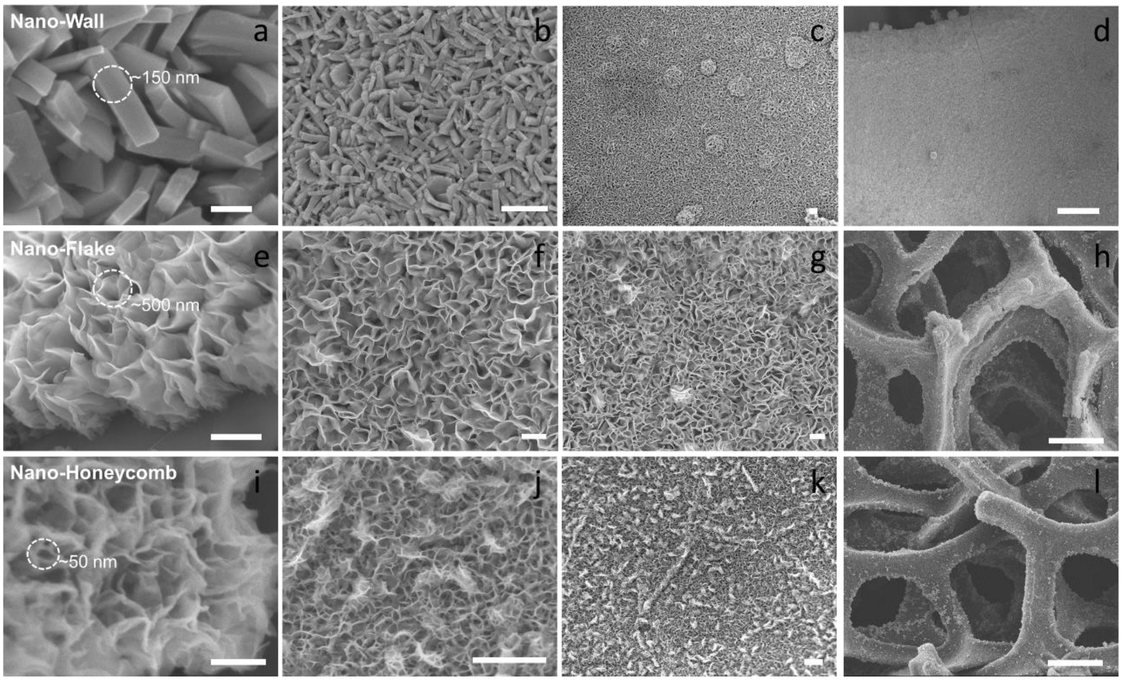

Supplementary Figure 1: Morphology evolution with the change in solution concentration. (a-d) GF-SnS NW electrodes at different magnification. (e-h) GF-SnS NF electrodes at different magnification. (i-l) GF-SnS NH electrodes at different magnification. Scale bars: in a,i 200 nm; d 10 μm; h,l 100 μm; others 1 μm.

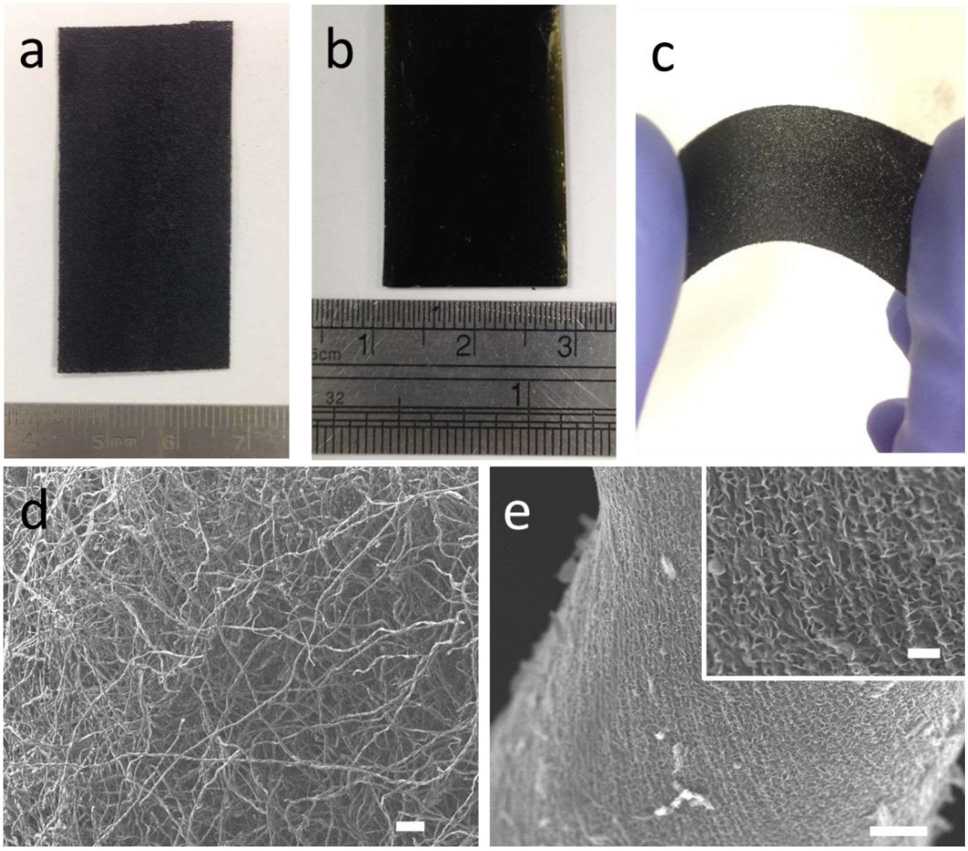

Supplementary Figure 2: Photos and SEM of the self-supported SnS electrodes on different substrates. (a) SnS nanosheets on Ni foam. (b) SnS nanosheets on ITO glass. (c) GF-SnS NH electrode that is bent by a small force to demonstrate the flexibility and lightweight of the electrode. (d,e) SEM images for SnS nanosheets on carbon cloth with scale bars 100 and 1 µm, respectively. Inset: an enlarged view (scale bar: 200 nm).

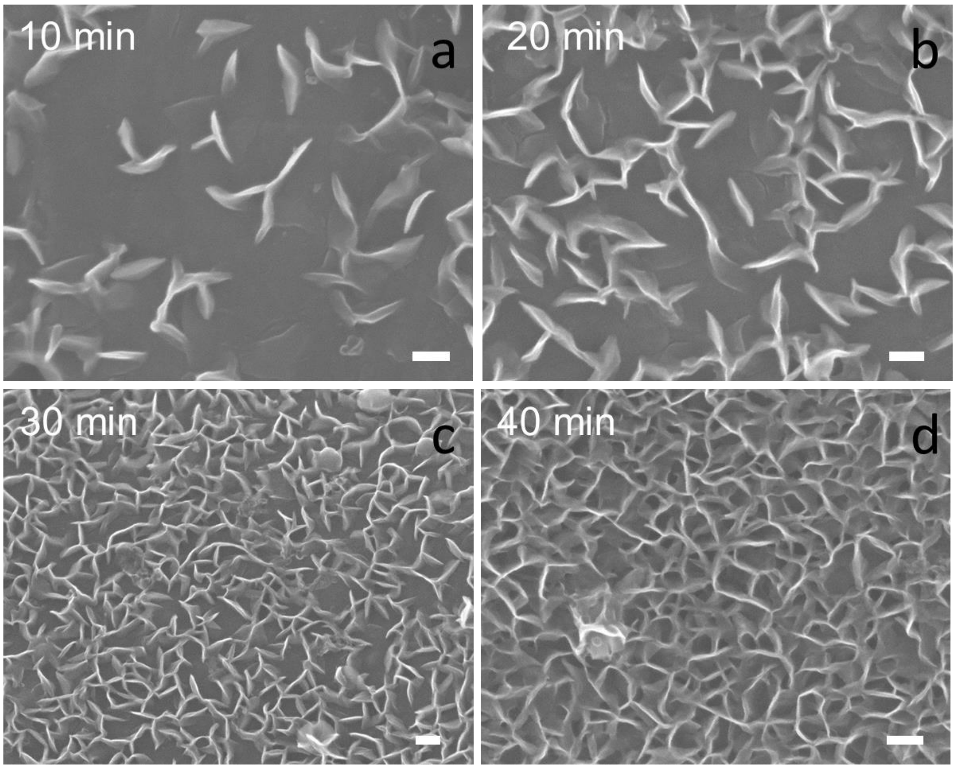

Supplementary Figure 3: Time-control experiments to reveal the morphology evolution of GF-SnS NH electrode. (a) SnS nucleates after hot bath reaction for 10 min. (b) for 20 min. (c) for 30 min. (d) for 40 min. The results may also indicate the chemical bonding between the NG and GF. Scale bars: 100 nm.

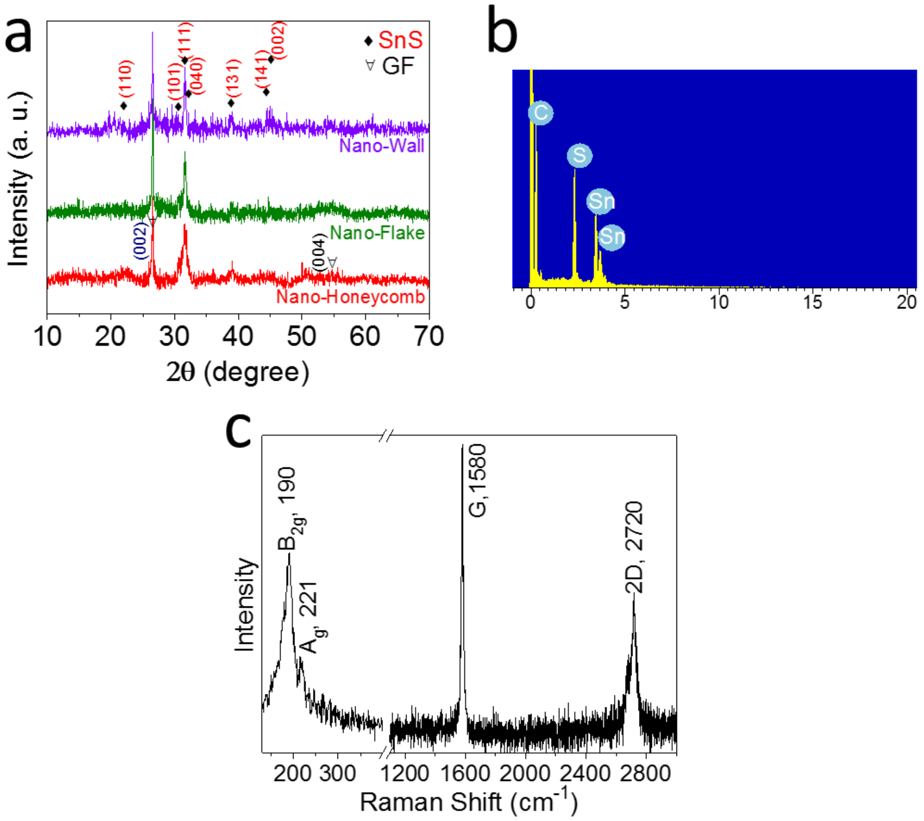

Supplementary Figure 4: Structure characterization of GF supported SnS nanosheet electrodes. (a) XRD patterns for the GF supported NW, NF, and NH electrodes. (b) EDS spectrum, which gives Sn:S ratio of nearly 1:1 for NH electrode. (c) Raman spectrum of NH electrode.

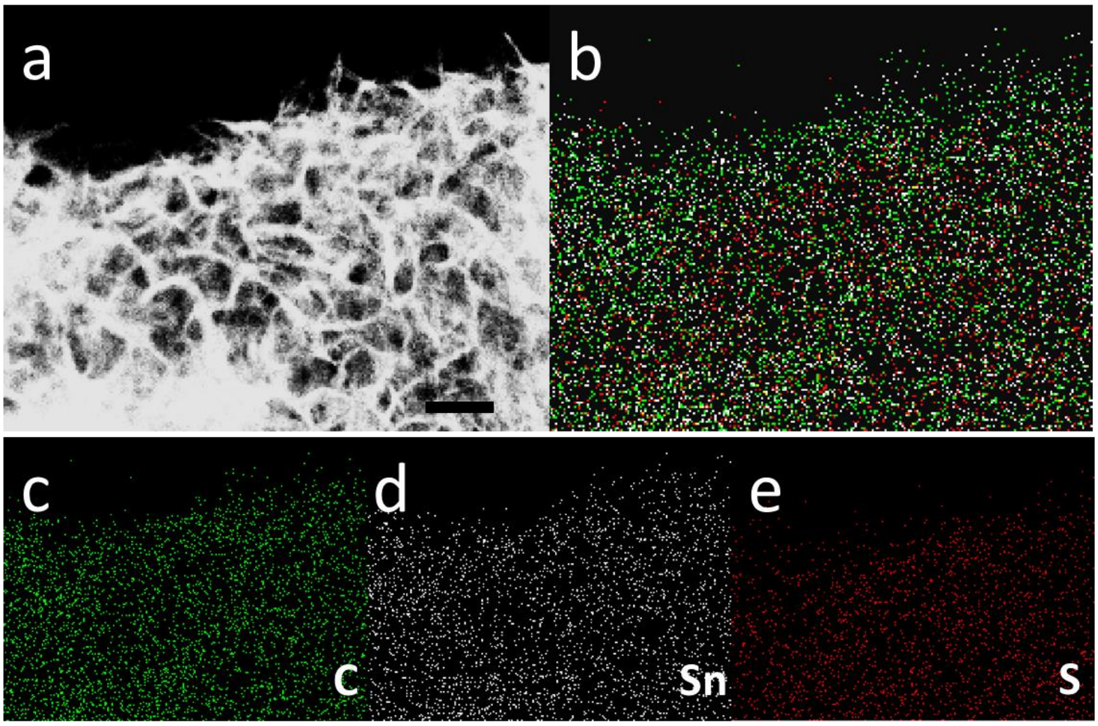

Supplementary Figure 5: TEM mapping of GF-SnS NH electrode. (a) Dark-field image of GF-SnS NH electrode. Scale bar: 100 nm. (b) Mixed EDS mapping of C (green), Sn (white), and S (red) elements. (c-e) EDS mapping of C, Sn, and S.

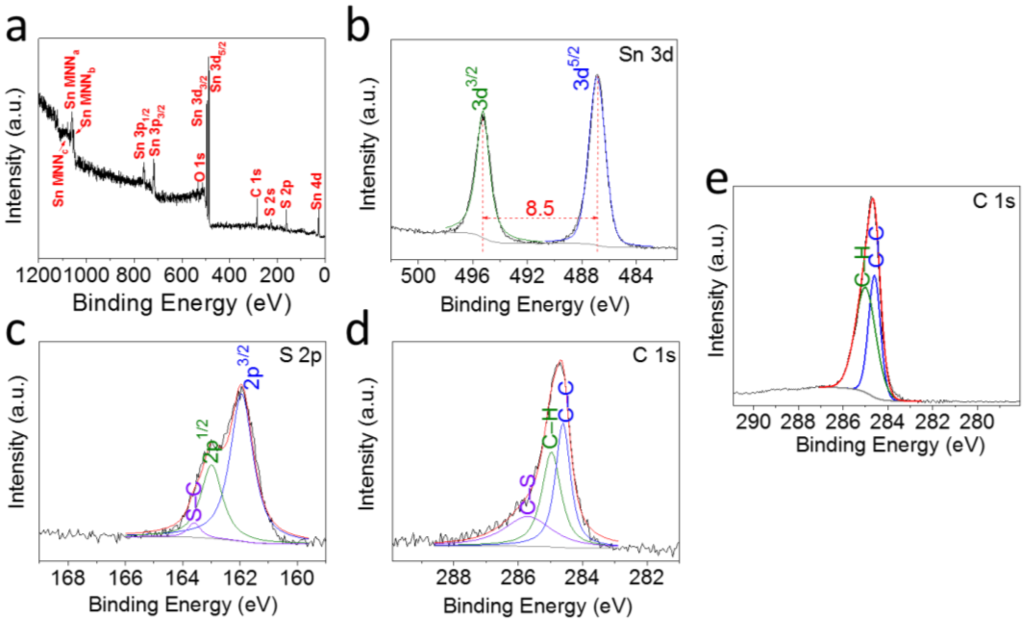

Supplementary Figure 6: Surface chemical bonding state of GF-SnS electrode. (a) XPS spectrum of GF-SnS NH electrode. (b-d) High-resolution XPS spectra of Sn 3d, S 2p, and C 1s in the GF-SnS NH electrode. (e) High-resolution XPS spectra of C 1s of pure GF as a comparison.

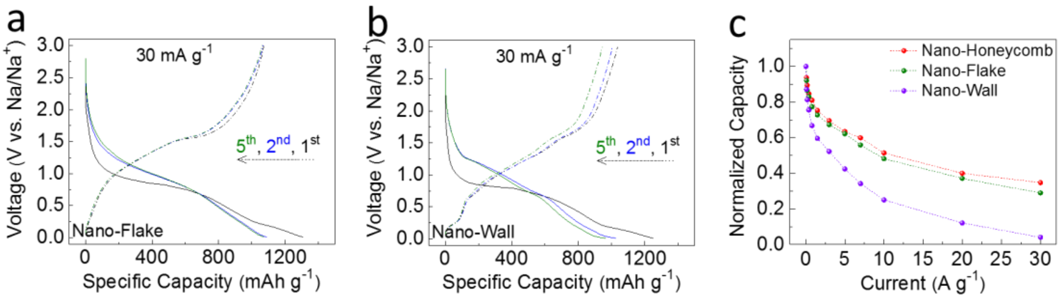

Supplementary Figure 7: Electrochemical property of the GF-SnS electrodes. (a,b) Galvanostatic charge/discharge profiles during the first 5 cycles of NF and NW electrodes at 30 mA g^{-1}. (c) Normalized rate capacities of GF-SnS electrodes at various current densities from 30 to 30,000 mA g^{-1}.

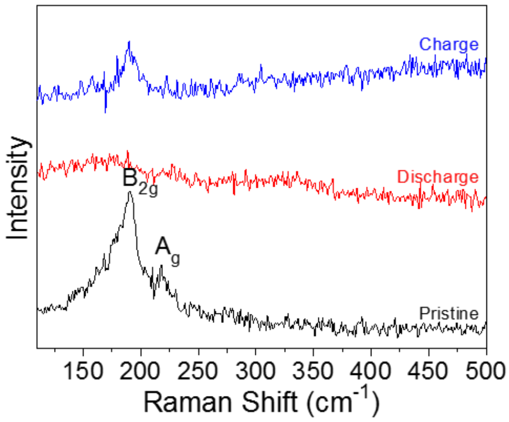

Supplementary Figure 8: Raman spectra of the SnS NH electrode at the pristine state, after first full discharge at 30 mA g^{-1}, and after first full charge at 30 mA g^{-1}. The electrode after discharge shows the elimination of B_{2g} peak suggesting the consumption of SnS. Afterwards, the reappeared broad peak after charging indicates the good reversibility of SnS nanostructures.

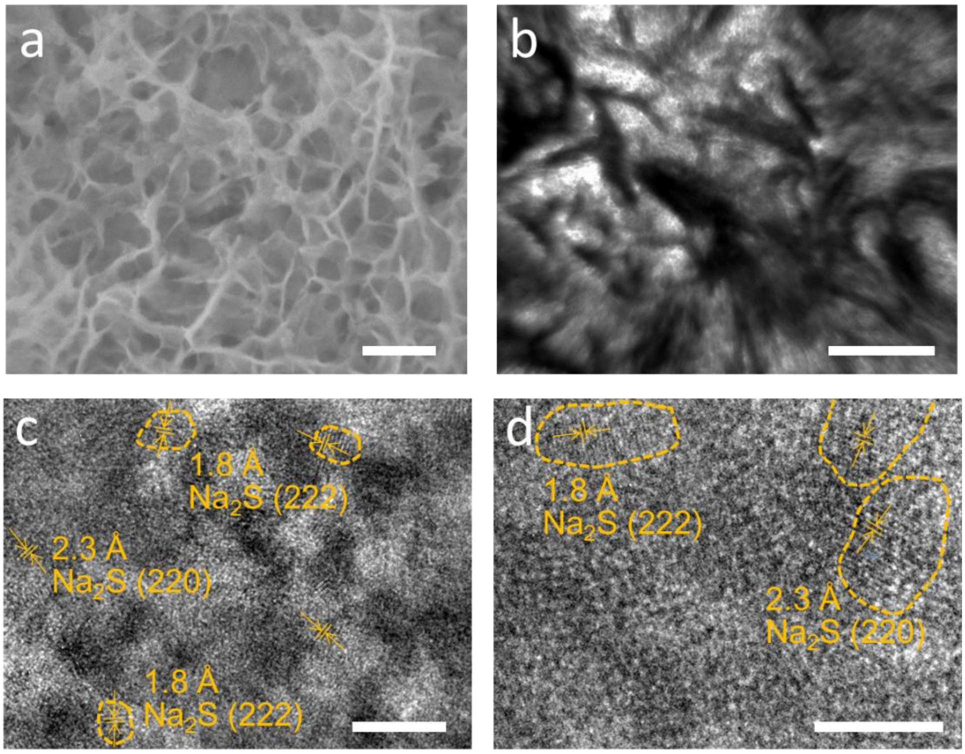

Supplementary Figure 9: Nanostructures after 200 cycles. Typical SEM (a) and TEM (b) images of NH electrode which show preserved the network structure after 200 cycles with scale bars 200, and 50 nm, respectively. Ex-situ HRTEM images of the electrode after full discharge: (c) for NH and (d) for NW electrodes with scale bars 5 nm. The appearance of Na_2S and disappearance of SnS crystal after full discharge, indicating that conversion and alloying happen simultaneously in the lower voltage region. Compared to the discharge products of NW electrode, the NH electrode present a more porous and finer particle after full discharge, which may be related with the different structure properties of the original NH and NW electrodes.

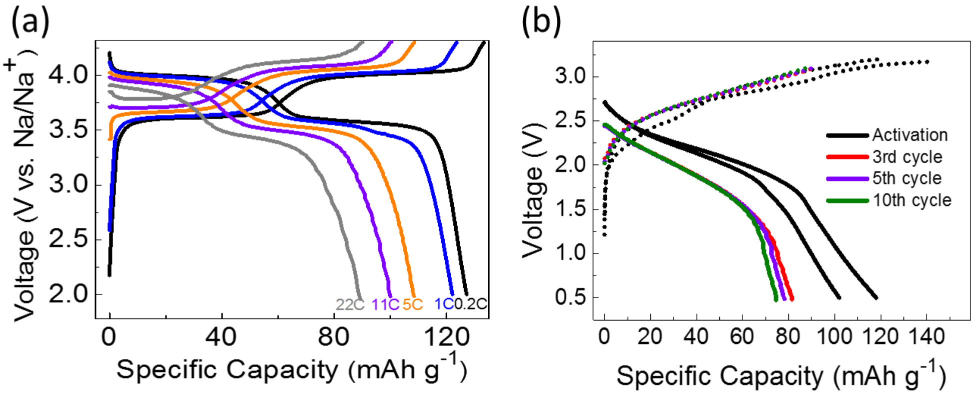

Supplementary Figure 10: Full cell demonstration. (a) Rate profiles of a $Na_3(VO)_2(PO_4)_2F$ nanocuboid cathode between 4.2 and 2.0 V. (b) Current full cell demonstration of $Na_3(VO)_2(PO_4)_2F$//SnS between 0.5 and 3.1 V at a current density of 0.1 A g^{-1} (black lines) for activation and 0.5 A g^{-1} for the following cycling. The $Na_3(VO)_2(PO_4)_2F$ cathode deliver a discharge capacity of 127 mAh g^{-1} at 0.2 C and is able to remain a high capacity of 88 mAh g^{-1} even at 22 C. When coupling $Na_3(VO)_2(PO_4)_2F$ with SnS electrode, the coin-type full-cell give a capacity of ca. 100 mAh g^{-1} at 0.1 A g^{-1} and ca. 80 mAh g^{-1} at 0.5 A g^{-1} (the specific capacity was calculated based on the mass of the $Na_3(VO)_2(PO_4)_2F$ cathode).

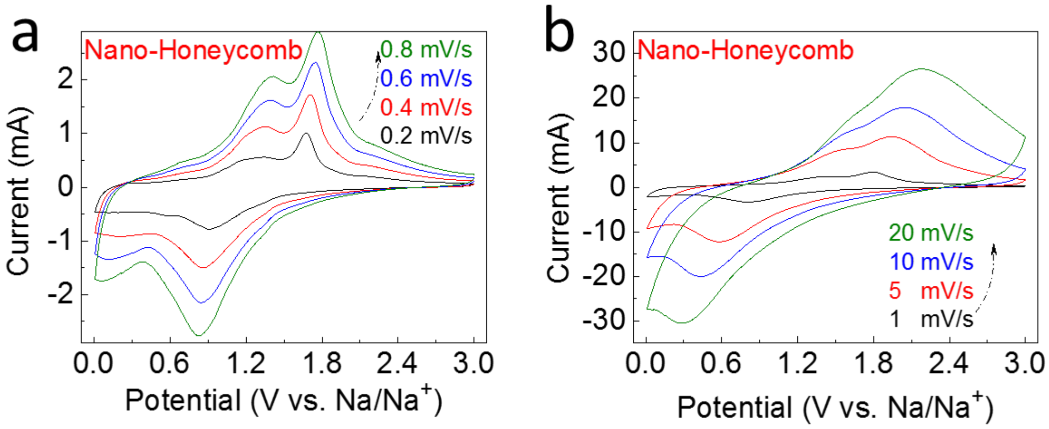

Supplementary Figure 11: CV curves of NH electrode for Na^+ storage at different scan rates.

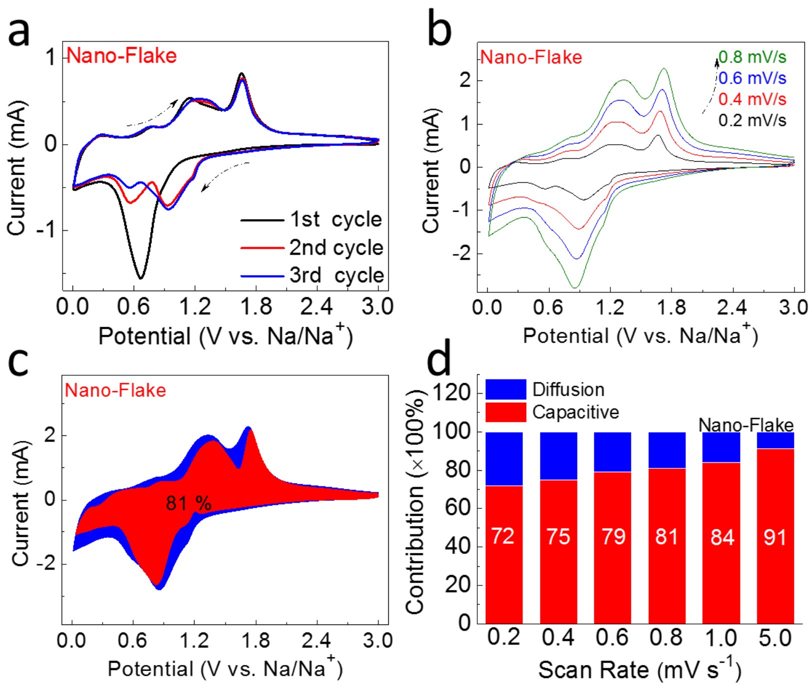

Supplementary Figure 12: Kinetics and quantitative analysis of the electrochemical mechanism of GF-SnS NF electrode. (a) CV curves of the first 3 cycles at a scan rate of 0.2 mV s^{-1}. (b) CV curves at various scan rate of 0.2, 0.4, 0.6, 0.8 mV s^{-1}. (c) Capacitive (red) and diffusion-controlled (blue) contribution to charge storage at 0.8 mV s^{-1}. (d) Normalized contribution ratio of capacitive (red) and diffusion-controlled (blue) capacities at different scan rates.

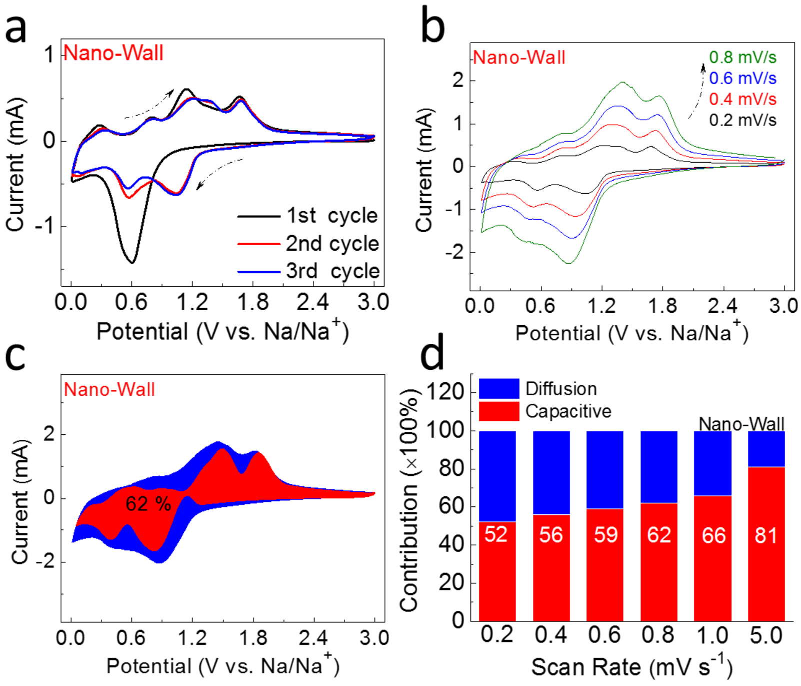

Supplementary Figure 13: Kinetics and quantitative analysis of the electrochemical mechanism of GF-SnS NW electrode. (a) CV curves of the first 3 cycles at a scan rate of 0.2 mV s^{-1}. (b) CV curves at various scan rate of 0.2, 0.4, 0.6, 0.8 mV s^{-1}. (c) Capacitive (red) and diffusion-controlled (blue) contribution to charge storage at 0.8 mV s^{-1}. (d) Normalized contribution ratio of capacitive (red) and diffusion-controlled (blue) capacities at different scan rates.

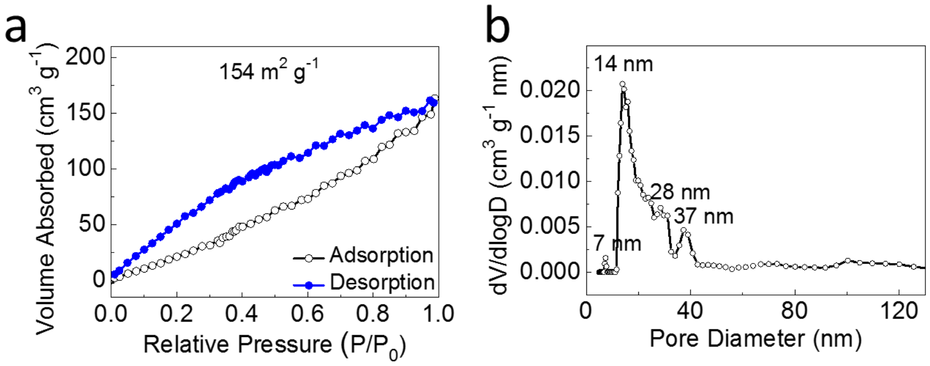

Supplementary Figure 14: (a,b) N_2 adsorption/desorption isotherm, and the corresponding pore size distribution of GF-SnS NH electrode. The SnS NH electrode deliver a specific surface area of ca 154 m^2 g^{-1}. The pore size distribution derived from BJH method shows peaks at 7, 14, 28, 37 nm, suggesting a highly mesoporous feature of the NH electrode.

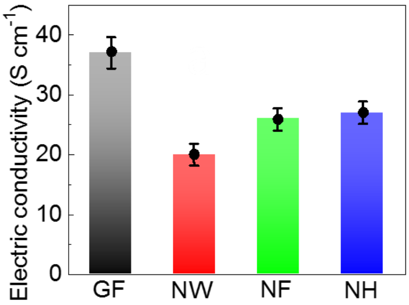

Supplementary Figure 15: Electric conductivity of GF, GF-SnS NW, NF, and NH films at the same pressing condition. The average conductivity was calculated over five specimens for each sample.

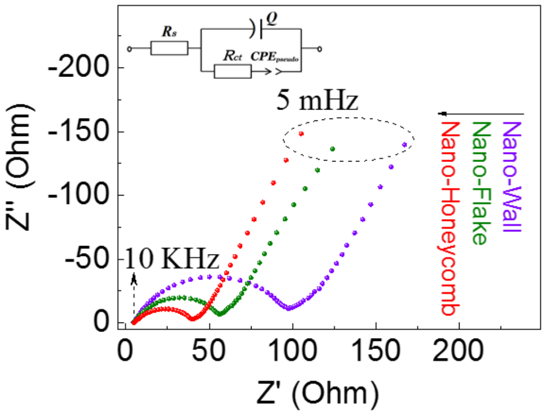

Supplementary Figure 16: Electrochemical AC impedance spectrum. At full-charged state after the first three cycles of GF-SnS electrodes. The resistance is simulated using inset equivalent circuit of $R_S(Q(R_{ct}CPE_{pseudo}))$[1], where R_S is the ohmic resistance of solution and electrodes, R_{ct} is the charge transfer resistance, Q is the double layer capacitance, and CPE_{pseudo} represents constant phase element ($Z_{CPE} = Y_0^{-1}(jw)^{-n}$, n is an empirical constant, ranging from 0 to 1). Simulations indicate that the charge transfer resistance of NW, NF, and NH electrodes are 103, 65, and 46 Ohm, respectively.

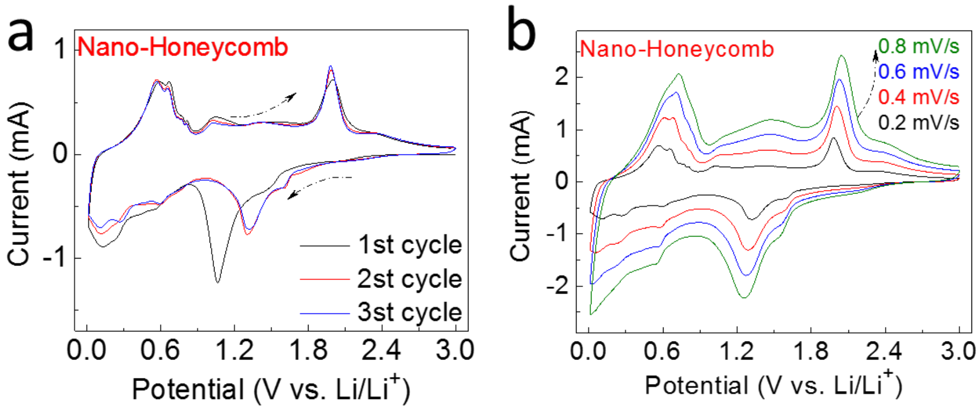

Supplementary Figure 17: CV curves of NF-SnS NH electrode for Li-ion battery. (a) CV curves of the first 3 cycles at a scan rate of 0.2 mV s^{-1}. (b) CV curves at various scan rate of 0.2, 0.4, 0.6, 0.8 mV s^{-1}.

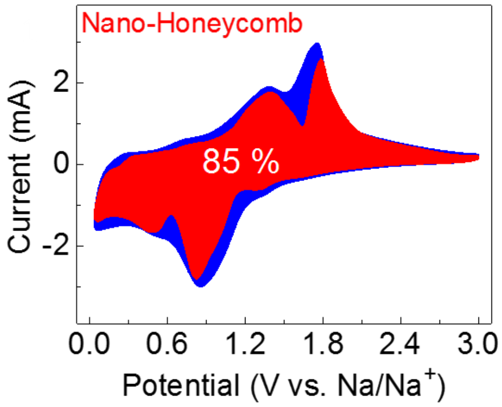

Supplementary Figure 18: Capacitive (red) and diffusion-controlled (blue) contribution to Na^+ charge storage of Ni foam supported NH SnS electrode at 0.8 mV s^{-1}, demonstrating similar property to the graphene foam-supported NH SnS electrode.

Supplementary Table 1: Three typical battery materials enhanced by extrinsic pseudocapacitance.

Mechanisms	Intercalation	Conversion	Alloying
Battery Materials (diffusion-controlled)[2]	Carbon allotropes, Nb_2O_5, TiO_2, $Li_4Ti_5O_{12}$, etc.	Fe_2O_3, Co_3O_4, V_2O_5, MnO_2, etc.	Si, Sn, Ge, etc.
With Extrinsic Pseudocapacitance[3-6]	$LiCoO_2$, etc.	V_2O_5, MoO_2, $Ni(OH)_2$, etc.	

Supplementary Table 2: A survey of electrochemical properties of anodes in sodium ion batteries.

Electrode description	Mass loading (mg cm^2)	Reversible capacity (mAh g^{-1})	Cycling stability [a] (%)	ICE (%) [c]	High rate capability (%)	Reference
GF-SnS NH [b] (this work)	1.0-1.2	1100 mAh g^{-1} at 30 mA g^{-1}	92 % after 200 cycles at 30 mA g^{-1}	81 %	420 mAh g^{-1} at 30 A g^{-1}	(this work)
Porous γ-Fe_2O_3@C nanocomposite	1.0	750 mAh g^{-1} at 200 mA g^{-1}	79 % after 200 cycles at 200 mA g^{-1}	78 %	340 mAh g^{-1} at 8 A g^{-1}	Supplementary References 7
Hollow carbon nanowire	Not provided	250 mAh g^{-1} at 50 mA g^{-1}	84 % after 400 cycles at 50 mA g^{-1}	51 %	150 mAh g^{-1} at 0.5 A g^{-1}	Supplementary References 8
Se-C composite	Not provided	485 mAh g^{-1} at 170 mA g^{-1}	70 % after 380 cycles at 170 mA g^{-1}	45 %	168 mAh g^{-1} at 3.4 A g^{-1}	Supplementary References 9
Micrometric Sb	1.4	600 mAh g^{-1} at 300 mA g^{-1}	96 % after 160 cycles at 300 mA g^{-1}	75 %	552 mAh g^{-1} at 2.4 A g^{-1}	Supplementary References 10
$Na_7V_4(P_2O_7)_4(PO_4)$-C nanorod	Not provided	40 mAh g^{-1} at 320 mA g^{-1}	80 % after 50 cycles at 320 mA g^{-1}		37 mAh g^{-1} at 1 A g^{-1}	Supplementary References 11
SnS	Not provided	520 mAh g^{-1} at 125 mA g^{-1}	65 % after 30 cycles at 125 mA g^{-1}	60 %	300 mAh g^{-1} at 1 A g^{-1}	Supplementary References 12
Sn-Al_2O_3 nanoparticle	Not provided	650 mAh g^{-1} at 85 mA g^{-1}	93 % after 40 cycles at 85 mA g^{-1}	65 %		Supplementary References 13
Porous $Li_4Ti_5O_{12}$	0.8-1.0	158 mAh g^{-1} at 18 mA g^{-1}			10 mAh g^{-1} at 5.3 A g^{-1}	Supplementary References 14
MoS_2 nanoflower	Not provided	350 mAh g^{-1} at 50 mA g^{-1}	140 % after 1500 cycles at 1 A g^{-1}		195 mAh g^{-1} at 10 A g^{-1}	Supplementary References 15
Pyrite FeS_2	Not provided	200 mAh g^{-1} at 200 mA g^{-1}	90 % after 20000 cycles at 1 A g^{-1}	75 %	170 mAh g^{-1} at 20 A g^{-1}	Supplementary References 16
TiO_2-C Nanorods	3.5	90 mAh g^{-1} at 1.65 A g^{-1}	90 % after 50 cycles at 1.65 A g^{-1}		53 mAh g^{-1} at 33 A g^{-1}	Supplementary References 17
$Li_4Ti_5O_{12}$-C nanowire	Not provided	168 mAh g^{-1} at 35 mA g^{-1}	97 % after 50 cycles at 35 mA g^{-1}		38 mAh g^{-1} at 17.5 A g^{-1}	Supplementary References 18
Li activated Ge nanowire	0.2212	350 mAh g^{-1} at 55.4 mA g^{-1}	97 % after 50 cycles at 55.4 mA g^{-1}	79 %	169 mAh g^{-1} at 3.69 A g^{-1}	Supplementary References 19
MoS_2-G	2.4	250 mAh g^{-1} at 25 mA g^{-1}	88 % after 20 cycles at 25 mA g^{-1}	75 %	200 mAh g^{-1} at 0.2 A g^{-1}	Supplementary References 20
SnS_2-G	1.0-1.5	610 mAh g^{-1} at 200 mA g^{-1}	63 % after 100 cycles at 200 mA g^{-1}	67 %	300 mAh g^{-1} at 4 A g^{-1}	Supplementary References 21
SnS_2-GO	Not provided	580 mAh g^{-1} at 50 mA g^{-1}	95 % after 50 cycles at 50 mA g^{-1}	67 %	350 mAh g^{-1} at 2 A g^{-1}	Supplementary References 22
Sb-C	3.0	610 mAh g^{-1} at 100 mA g^{-1}	94 % after 100 cycles at 100 mA g^{-1}	85 %	309 mAh g^{-1} at 2 A g^{-1}	Supplementary References 23
SnS_2-rGO	1.67	550 mAh g^{-1} at 1 A g^{-1}	91 % after 400 cycles at 1 A g^{-1}	79 %	570 mAh g^{-1} at 2 A g^{-1}	Supplementary References 24
SnS-C	1.2	490 mAh g^{-1} at 500 mA g^{-1}	88 % after 50 cycles at 500 mA g^{-1}	63 %	300 mAh g^{-1} at 5 A g^{-1}	Supplementary References 25
CoS_2-MWCNT	Not provided	650 mAh g^{-1} at 100 mA g^{-1}	88 % after100 cycles at 100 mA g^{-1}	93 %	525 mAh g^{-1} at 0.8 A g^{-1}	Supplementary References 26
NiS-PEDOT	1.0	450 mAh g^{-1} at	60 % after 50 cycles	83 %	300 mAh g^{-1}	Supplementary

		600 mA g^{-1}	at 600 mA g^{-1}		at 1.2 A g^{-1}	References 27
SnO_2-G	Not provided	650 mAh g^{-1} at 20 mA g^{-1}	95 % after 100 cycles at 20 mA g^{-1}	31 %	150 mAh g^{-1} at 0.64 A g^{-1}	Supplementary References 28
MoS_2 nanosheet	1.2	550 mAh g^{-1} at 80 mA g^{-1}	64 % after 100 cycles at 80 mA g^{-1}	42 %	300 mAh g^{-1} at 0.32 A g^{-1}	Supplementary References 29
MoS_2-C	Not provided	600 mAh g^{-1} at 67 mA g^{-1}	87 % after 50 cycles at 67 mA g^{-1}	45 %	380 mAh g^{-1} at 1.34 A g^{-1}	Supplementary References 30
Sn-SnS-C composite	Not provided	450 mAh g^{-1} at 100 mA g^{-1}	87% after 150 cycles at 100 mA g^{-1}	62 %	350 mAh g^{-1} at 0.8 A g^{-1}	Supplementary References 31
Sb-C	Not provided	635 mAh g^{-1} at 200 mA g^{-1}	94% after 100 cycles at 200 mA g^{-1}	64 %	310 mAh g^{-1} at 3 A g^{-1}	Supplementary References 32
SnS-rGO	2.4	457 mAh g^{-1} at 20 mA g^{-1}	94% after 100 cycles at 100 mA g^{-1}	56 %	240 mAh g^{-1} at 0.4 A g^{-1}	Supplementary References 33
SnS-C	2.0	544 mAh g^{-1} at 100 mA g^{-1}	98% after 80 cycles at 100 mA g^{-1}	66 %	450 mAh g^{-1} at 0.8 A g^{-1}	Supplementary References 34
SnSb-C	Not provided	485 mAh g^{-1} at 100 mA g^{-1}	81% after 50 cycles at 100 mA g^{-1}	75 %	295 mAh g^{-1} at 1 A g^{-1}	Supplementary References 35
MoS_2-G	1.0	440 mAh g^{-1} at 20 mA g^{-1}	63% after 100 cycles at 20 mA g^{-1}	41 %	352 mAh g^{-1} at 0.64 A g^{-1}	Supplementary References 36
Sn-CNT	1.2	600 μAh cm^{-2} at 50μA cm^{-2}	92% after 100 cycles at 50μA cm^{-2}	81 %	300 mAh g^{-1} at 1 mA cm^{-2}	Supplementary References 37
SnS_2-G	1.0	725 mAh g^{-1} at 20 mA g^{-1}	89% after 60 cycles at 20 mA g^{-1}	52 %	463 mAh g^{-1} at 0.64 A g^{-1}	Supplementary References 38
Sn-C	0.5	300 mAh g^{-1} at 20 mA g^{-1}	60% after 15 cycles at 20 mA g^{-1}	40 %	20 mAh g^{-1} at 1 A g^{-1}	Supplementary References 39
Ni-Sn-C	0.5	405 mAh g^{-1} at 50 mA g^{-1}	60% after 150 cycles at 50 mA g^{-1}	78 %		Supplementary References 40
Sb_2S_3-rGO	1.5-1.8	650 mAh g^{-1} at 50 mA g^{-1}	97% after 50 cycles at 50 mA g^{-1}	68 %	520 mAh g^{-1} at 3 A g^{-1}	Supplementary References 41
MoS_2-CNT	1.5	496 mAh g^{-1} at 200 mA g^{-1}	85% after 80 cycles at 200 mA g^{-1}	67 %	300 mAh g^{-1} at 0.5 A g^{-1}	Supplementary References 42
SnS_2-rGO	2.3	649 mAh g^{-1} at 100 mA g^{-1}	89% after 400 cycles at 800 mA g^{-1}	64 %	337 mAh g^{-1} at 12.8 A g^{-1}	Supplementary References 43
SnS-G	1.0	500 mAh g^{-1} at 810 mA g^{-1}	94% after 250 cycles at 810 mA g^{-1}	70 %	308 mAh g^{-1} at 7.29 A g^{-1}	Supplementary References 44
Sb_2S_3	Not provided	835 mAh g^{-1} at 50 mA g^{-1}	92% after 100 cycles at 200 mA g^{-1}	73 %	553.1 mAh g^{-1} at 2 A g^{-1}	Supplementary References 45

[a] The cycling stability is calculated by $C_n/C_{max} \times 100\%$, where C_n is the discharge capacity at the n cycle, C_{max} is the maximum discharge capacity after the first 3 cycles; [b] All the capacity values were all calculated based on the active material SnS only and include no weight of current collector (GF); [c] Initial coulombic efficiency (* 100%).

Supplementary Note 1

Discussion on growth mechanism of SnS nanosheets:

$$CH_3C(S)NH_2+H_2O\rightarrow CH_3C(O)NH_2+H_2S \quad (1)$$

$$Sn^{2+}+H_2S\rightarrow SnS+2H^+ \quad (2)$$

First, sulfur source thioacetamide and hydrated tin chloride could homogeneously disperse in ethanol, then $CH_3C(S)NH_2$ reacts with trace water from $SnCl_2{\cdot}2H_2O$ as shown in Eq. (1)[46]. Furthermore, H_2S was produced slowly and *in-situ* metathesis reacted with Sn^{2+} to form sulfides. In addition to strong crystal anisotropy of the layered metal chalcogenide, the oriented growth of SnS nanocrystals may be closely related to the GF backbone and trace water in the system. During the hot bath reaction, the Sn^{2+} favorably nucleates onto the surface of GF with the help of ethanol, where bonding between the particles reduces the overall energy. Meanwhile, the trace water molecules, which is surrounded by ethanol molecules with hydrogen bonds[46], would array in chains and drift close to the GF backbone. It is believed that the chain texture of water molecules is preferential to the oriented growth of SnS nanosheets. An implication can be found by the structure evolution during the reactions in Supplementary Figure 3. On the other hand, the numerous bubbles, originating from ethanol at appropriate temperature, create a continuous path from the GF substrate to the surface of bubbles[47]. In other words, the bubbles act not only as a dynamic template for the oriented growth of the SnS nanocrystals, but also as a gas blocker for the formation of 3D porous nanoarchitectures. Herein, strategy of concentration control was employed to facilitate the assembly of preferred SnS nanostructures. As the decrease of the precursor concentration, the nucleation rate decreases, thus results in smaller nucleation particle sizes; Moreover, the presence of more electrolytes could promote instant 3D coagulation and aggregation for the growth of direction coordinated structures, thus facilitating the formation of thicker nano-wall SnS aggregates[48-50].

Supplementary Note 2

Discussion on structure characterization of SnS nanosheets:

The X-ray diffraction (XRD) patterns of the samples with are presented in Supplementary Figure 4a. The XRD peaks can be indexed to the graphitic carbon (JCPDS 75−1621) and orthorhombic SnS (JCPDS 39–0354). Obviously, the (111) peak of orthorhombic SnS in NH shows a broadening feature compared with that of NF and NW, revealing a finer grains character of the SnS NH crystallites. The strong (111) peak for SnS is the only relatively obvious peak, revealing the high preferential orientation along the (111) plane of the as-prepared samples, which is coincidence with the reported SnS results[51, 52]. Roughly, The average crystallite size calculated from the (111) diffraction peak by the Scherrer equation is around 30 nm for SnS NW electrode, while 15 and 8 nm for NF and NH electrodes, respectively. Meanwhile, from the XRD pattern, it can be seen that no impurities are detected in the samples, which was further confirmed by the EDS analysis (Supplementary Figure 4b and 5). The formation of pure tin sulfides without oxides and hydrates may be related to the weak acidic condition due to the hydrolysis and metathesis reactions. Raman spectrum of the as-prepared GF-SnS electrode (Supplementary Figure 4c) further confirms these results. Two distinct peaks at 190 and 221 cm^{-1} can be assigned to the B_{2g} and A_g mode of SnS. B_{2g} modes were detected possibly due to the high orientation of the as-synthesized SnS nanosheets. The surface chemical bonding state of GF-SnS electrode is also detected by X-ray photoelectron spectroscopy (XPS) in Supplementary Figure 6. The binding energies of the analyzed XPS data were calibrated by pure Au. The binding energies located at 495.3 (Sn $3d_{3/2}$) and 486.8 eV (Sn $3d_{5/2}$) are characteristic peaks of Sn^{2+} in SnS. No evidence of Sn^{4+} is found. The ratio of the integral area for Sn 3d to S 2p is about 1:1.02, which is consistent with the EDS results. More importantly, the existence of C-S bonds in GF-SnS is confirmed by spectra of both S 2p (163.7 ev) and C 1s (285.7 ev). As a comparison, the related peak was not found for the pure GF samples. These results suggests that the SnS might be chemically bonded with the GF matrix besides physical adsorption[53].

Supplementary References

1. Kim, J. W., Augustyn, V. & Dunn, B. The effect of crystallinity on the rapid pseudocapacitive response of Nb2O5. *Adv. Energy Mater.* **2**, 141-148 (2012).
2. Armand, M. & Tarascon, J. M. Building better batteries. *Nature* **451**, 652-657 (2008).
3. Kim, H. S., Cook, J. B., Tolbert, S. H. & Dunn, B. The development of pseudocapacitive properties in nanosized-MoO2. *J. Electrochem. Soc.* **162**, A5083-A5090 (2015).
4. Okubo, M. *et al.* Nanosize effect on high-rate Li-ion intercalation in LiCoO2 electrode. *J. Am. Chem. Soc.* **129**, 7444-7452 (2007).
5. Sathiya, M., Prakash, A., Ramesha, K., Tarascon, J. M. & Shukla, A. V2O5-anchored carbon nanotubes for enhanced electrochemical energy storage. *J. Am. Chem. Soc.* **133**, 16291-16299 (2011).
6. Augustyn, V., Simon, P. & Dunn, B. Pseudocapacitive oxide materials for high-rate electrochemical energy storage. *Energy Environ. Sci.* **7**, 1597-1614 (2014).
7. Zhang, N. *et al.* 3D porous γ-Fe2O3@C nanocomposite as high-performance anode material of Na-ion batteries. *Adv. Energy Mater.* **5**, 201401123 (2015).
8. Cao, Y. *et al.* Sodium ion insertion in hollow carbon nanowires for battery applications. *Nano Lett.* **12**, 3783-3787 (2012).
9. Luo, C. *et al.* Selenium@mesoporous carbon composite with superior lithium and sodium storage capacity. *Acs Nano* **7**, 8003-8010 (2013).
10. Darwiche, A. *et al.* Better cycling performances of bulk Sb in Na-ion batteries compared to Li-ion systems: an unexpected electrochemical mechanism. *J. Am. Chem. Soc.* **134**, 20805-20811 (2012).
11. Deng, C., Zhang, S. & Wu, Y. Hydrothermal-assisted synthesis of the Na7V4(P2O7)4(PO4)/C nanorod and its fast sodium intercalation chemistry in aqueous rechargeable sodium batteries. *Nanoscale* **7**, 487-491 (2014).
12. Dutta, P. K., Sen, U. K. & Mitra, S. Excellent electrochemical performance of tin monosulphide (SnS) as a sodium-ion battery anode. *RSC Adv.* **4**, 43155-43159 (2014).
13. Han, X. *et al.* Atomic-layer-deposition oxide nanoglue for sodium ion batteries. *Nano Lett.* **14**, 139-147 (2014).
14. Hasegawa, G. *et al.* Hierarchically porous Li4Ti5O12 anode materials for Li- and Na-ion batteries: effects of nanoarchitectural design and temperature dependence of the rate capability. *Adv. Energy Mater.* **5**, 201400730 (2015).
15. Hu, Z. *et al.* MoS2 nanoflowers with expanded interlayers as high-performance anodes for sodium-ion batteries. *Angewandte Chemie* **126**, 13008-13012 (2014).
16. Hu, Z. *et al.* Pyrite FeS2for high-rate and long-life rechargeable sodium batteries. *Energy Environ. Sci.* **8**, 1309-1316 (2015).
17. Kim, K. T. *et al.* Anatase titania nanorods as an intercalation anode material for rechargeable sodium batteries. *Nano Lett.* **14**, 416-422 (2014).
18. Kim, K.-T. *et al.* Carbon-coated Li4Ti5O12 nanowires showing high rate capability as an anode material for rechargeable sodium batteries. *Nano Energy* **12**, 725-734 (2015).
19. Kohandehghan, A. *et al.* Activation with Li enables facile sodium storage in germanium. *Nano Lett.* **14**, 5873-5882 (2014).
20. David, L., Bhandavat, R. & Singh, G. MoS2/graphene composite paper for sodium-ion battery electrodes. *ACS Nano* **8**, 1759-1770 (2014).
21. Liu, Y. *et al.* Exfoliated-SnS2 restacked on graphene as a high-capacity, high-rate, and long-cycle life anode for sodium ion batteries. *Nanoscale* **7**, 1325-1332 (2015).
22. Prikhodchenko, P. V. *et al.* Nanocrystalline tin disulfide coating of reduced graphene oxide produced by the peroxostannate deposition route for sodium ion battery anodes. *J. Mater. Chem. A* **2**, 8431 (2014).
23. Qian, J. *et al.* High capacity Na-storage and superior cyclability of nanocomposite Sb/C anode for Na-ion batteries. *Chem Commun (Camb)* **48**, 7070-7072 (2012).
24. Qu, B. *et al.* Layered SnS2-reduced graphene oxide composite--a high-capacity, high-rate, and long-cycle life sodium-ion battery anode material. *Adv. Mater.* **26**, 3854-3859 (2014).
25. Choi, S. H. & Kang, Y. C. Aerosol-assisted rapid synthesis of SnS-C composite microspheres as anode material for Na-ion batteries. *Nano Research* **8**, 1595-1603 (2015).
26. Shadike, Z., Cao, M. H., Ding, F., Sang, L. & Fu, Z. W. Improved electrochemical performance of CoS2-MWCNT nanocomposites for sodium-ion batteries. *Chem Commun (Camb)* **51**, 10486-10489 (2015).
27. Shang, C. *et al.* A Ni3S2-PEDOT monolithic electrode for sodium batteries. *Electrochem. Commun.* **50**, 24-27 (2015).
28. Su, D., Ahn, H. J. & Wang, G. SnO2@graphene nanocomposites as anode materials for Na-ion batteries with superior electrochemical performance. *Chem Commun (Camb)* **49**, 3131-3133 (2013).
29. Su, D., Dou, S. & Wang, G. Ultrathin MoS2 nanosheets as anode materials for sodium-ion batteries with superior

performance. *Adv. Energy Mater.* **5**, 201401205 (2015).
30. Wang, J. *et al.* An advanced MoS2 /carbon anode for high-performance sodium-ion batteries. *Small* **11**, 473-481 (2015).
31. Wu, L. *et al.* A Sn–SnS–C nanocomposite as anode host materials for Na-ion batteries. *J. Mater. Chem. A* **1**, 7181 (2013).
32. Wu, L. *et al.* Electrochemical properties and morphological evolution of pitaya-like Sb@C microspheres as high-performance anode for sodium ion batteries. *J. Mater. Chem. A* **3**, 5708-5713 (2015).
33. Wu, L. *et al.* Improved sodium-storage performance of stannous sulfide@reduced graphene oxide composite as high capacity anodes for sodium-ion batteries. *J. Power Sources* **293**, 784-789 (2015).
34. Wu, L. *et al.* A tin(ii) sulfide–carbon anode material based on combined conversion and alloying reactions for sodium-ion batteries. *J. Mater. Chem. A* **2**, 16424-16428 (2014).
35. Xiao, L. *et al.* High capacity, reversible alloying reactions in SnSb/C nanocomposites for Na-ion battery applications. *Chem Commun (Camb)* **48**, 3321-3323 (2012).
36. Xie, X., Ao, Z., Su, D., Zhang, J. & Wang, G. MoS2/graphene composite anodes with enhanced performance for sodium-ion batteries: the role of the two-dimensional heterointerface. *Adv. Funct. Mater.* **25**, 1393-1403 (2015).
37. Xie, X. *et al.* Sn@CNT nanopillars grown perpendicularly on carbon paper: A novel free-standing anode for sodium ion batteries. *Nano Energy* **13**, 208-217 (2015).
38. Xie, X. *et al.* SnS2 nanoplatelet@graphene nanocomposites as high-capacity anode materials for sodium-ion batteries. *Chem. Asian J.* **9**, 1611-1617 (2014).
39. Xu, Y., Zhu, Y., Liu, Y. & Wang, C. Electrochemical performance of porous carbon/tin composite anodes for sodium-ion and lithium-ion batteries. *Adv. Energy Mater.* **3**, 128-133 (2013).
40. Liu, Y. H. *et al.* Tin-coated viral nanoforests as sodium-ion battery anodes. *Acs Nano* **7**, 3627-3634 (2013).
41. Yu, D. Y. *et al.* High-capacity antimony sulphide nanoparticle-decorated graphene composite as anode for sodium-ion batteries. *Nat. Commun.* **4**, 2922 (2013).
42. Zhang, S. *et al.* Growth of ultrathin MoS(2) nanosheets with expanded spacing of (002) plane on carbon nanotubes for high-performance sodium-ion battery anodes. *ACS Appl. Mater. Interfaces* **6**, 21880-21885 (2014).
43. Zhang, Y. *et al.* Few-layered SnS2 on few-layered reduced graphene oxide as Na-ion battery anode with ultralong cycle life and superior rate capability. *Adv. Funct. Mater.* **25**, 481-489 (2015).
44. Zhou, T. *et al.* Enhanced sodium-ion battery performance by structural phase transition from two-dimensional hexagonal-SnS2 to orthorhombic-SnS. *ACS Nano* **8**, 8323-8333 (2014).
45. Zhu, Y. *et al.* High rate capability and superior cycle stability of a flower-like Sb2S3 anode for high-capacity sodium ion batteries. *Nanoscale* **7**, 3309-3315 (2014).
46. McMurry, J. *Organic Chemistry*, 8 edn. Brooks/Cole (2012).
47. Shin, H. C., Dong, J. & Liu, M. Nanoporous structures prepared by an electrochemical deposition process. *Adv. Mater.* **15**, 1610-1614 (2003).
48. Gursky, J. A. *et al.* Particle−particle interactions between layered double hydroxide nanoparticles. *J. Am. Chem. Soc.* **128**, 8376-8377 (2006).
49. Ma, J. M. *et al.* Designable fabrication of flower-like SnS2 aggregates with excellent performance in lithium-ion batteries. *RSC Advances* **2**, 3615-3617 (2012).
50. Xu, Z. P., Stevenson, G., Lu, C.-Q. & Lu, G. Q. Dispersion and size control of layered double hydroxide nanoparticles in aqueous solutions. *J. Phys. Chem. B* **110**, 16923-16929 (2006).
51. Su, H., Xie, Y., Xiong, Y., Gao, P. & Qian, Y. Preparation and morphology control of rod-like nanocrystalline tin sulfides via a simple ethanol thermal route. *J. Solid State Chem.* **161**, 190-196 (2001).
52. Gou, X.-L., Chen, J. & Shen, P.-W. Synthesis, characterization and application of SnSx (x=1, 2) nanoparticles. *Mater. Chem. Phys.* **93**, 557-566 (2005).
53. Chen, C. *et al.* Na(+) intercalation pseudocapacitance in graphene-coupled titanium oxide enabling ultra-fast sodium storage and long-term cycling. *Nat. Commun.* **6**, 6929 (2015).
